# Origin of Life: The Point of No Return

**DOI:** 10.3390/life10110269

**Published:** 2020-11-03

**Authors:** Dimiter Kunnev

**Affiliations:** Department of Oral Biology, University at Buffalo, Buffalo, NY 14263, USA; dkunnev@buffalo.edu

**Keywords:** origin of life, definition of life, Darwinian evolution, biological evolution, prebiotic evolution, chemical evolution, genetic code, RNA-peptide world, RNA world, metabolism-first, replicator-first, first life, *Terra Darwinia*, first organism

## Abstract

Origin of life research is one of the greatest scientific frontiers of mankind. Many hypotheses have been proposed to explain how life began. Although different hypotheses emphasize different initial phenomena, all of them agree around one important concept: at some point, along with the chain of events toward life, Darwinian evolution emerged. There is no consensus, however, how this occurred. Frequently, the mechanism leading to Darwinian evolution is not addressed and it is assumed that this problem could be solved later, with experimental proof of the hypothesis. Here, the author first defines the minimum components required for Darwinian evolution and then from this standpoint, analyzes some of the hypotheses for the origin of life. Distinctive features of Darwinian evolution and life rooted in the interaction between information and its corresponding structure/function are then reviewed. Due to the obligatory dependency of the information and structure subject to Darwinian evolution, these components must be locked in their origin. One of the most distinctive characteristics of Darwinian evolution in comparison with all other processes is the establishment of a fundamentally new level of matter capable of evolving and adapting. Therefore, the initiation of Darwinian evolution is the “point of no return” after which life begins. In summary: a definition and a mechanism for Darwinian evolution are provided together with a critical analysis of some of the hypotheses for the origin of life.

*“All my life I have wondered about the possibility of life elsewhere. What would it be like? Of what would it be made? …Is extraterrestrial life, if it exists, based on the same organic molecules as life on Earth? Do the beings of other worlds look much like life on Earth? … What else is possible? The nature of life on Earth and the search for life elsewhere are two sides of the same question - the search for who we are.” Carl Sagan’s Cosmos*

## 1. Introduction

What distinguishes a living entity from matter that is not alive? How did life originate? These questions have accompanied us through human history. In searching for the first living cell, at some moment, a proto-cell(s) capable of evolving should emerge. The newly established proto-cell must have a relatively simple structure with heredity and a primordial metabolism [1,2]. However, the process leading to the formation of a proto-cell must consist of a multi-step process that begins with the precursors of nucleic acids, peptides, lipids, and energy followed by polymer interactions and enzymatic metabolic reactions. Based on our understanding of evolution, we can picture a scenario where multiple variants of proto-cells initially emerged but most adapted, survived, and evolved as the Last Universal Common Ancestor (LUCA) [2,3]. An obvious question is “What are the critical steps for living matter to emerge?” The formation of RNA and peptides depends on the previous steps of the prebiotic synthesis of nucleotides and amino acids. Polymer interactions and the emergence of sustained proto-cellular structure depend on previously synthesized polymers made of monomers; therefore, there is no specific single event that is recognized to have paramount importance for the beginning of life. Is it possible, therefore, to find a boundary between chemistry and biology if we look at all the events leading up to LUCA? According to Jack Szostak and other leading scientists, this question is not in the scope of origin of life research since it does not provide the basis for associated experiments and therefore eliminates the need to define a transition from chemistry to biology [4,5]. However, if we investigate the chain of events from the first polymers to the formation of the first proto-cells, there must be a moment where heredity starts to play a significant role. The establishment of heredity defines the emergence of adaptation and diversification toward more complex structures, i.e., Darwinian evolution. All events that occur before this moment are governed only by environmental conditions, where more complicated structures are synthesized simply by chance with no “recollection” of previously existing ones. The forces of degradation could roll back the initial state. Once Darwinian evolution is established, new forms and structures could be synthesized at a higher rate due to the existence of the mechanism of heredity. From this perspective, the initiation of Darwinian evolution defines a “point of no return”. Before this moment, any polymer complex would be assembled and disassembled throughout every cycle of events with the same probability. After this moment, the synthesis of more stable complexes occurs with increasing probability. Prior to the “point of no return”, if some combination of molecular interactions achieved higher stability by chance, it would fail to repeat this “success” with a frequency higher than the original occurrence. The development of Darwinian evolution may not have been a sudden process; it would have involved many trials and errors but would have made an enormous difference after it was established. Before and after Darwinian evolution’s “point of no return”, matter behaved differently as it obtained the ability to advance and adapt to new environments. The current paper advocates the premise that the origin of Darwinian evolution and the origin of life are inextricably bound and cannot be separated, therefore any reliable hypothesis for the origin of life must include a mechanism for the formation of Darwinian evolution. With this understanding, important conclusions will be presented which may be useful to anyone in the field of origin of life research.

## 2. The Mechanism of Darwinian Evolution

The mechanism of Darwinian evolution is the key to understanding the initial complex that triggers the origin of life. Darwinian evolution is a well understood phenomenon in terms of genetic variations, natural selection, heredity, and mathematical modeling, as has been shown many times [6,7,8,9,10,11,12,13]. The task here is to define the minimum number of components working on the molecular level necessary for selection to occur, so these might be utilized in the further origin of life research.

**Figure 1:**
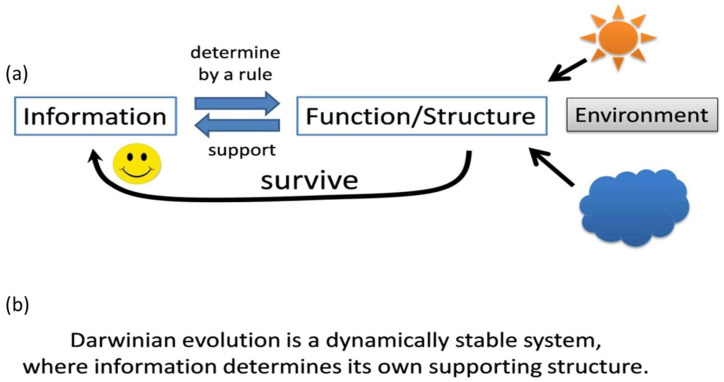
Darwinian evolution: mechanism of action. (**a**) Darwinian evolution exists through the interrelationship of an information carrier and a structural component. The information carrier holds the heredity attributes of the system and it is able to preserve changes to the system. The supporting structural component is coupled to the information component through a rule that maps changes in the information carrier to changes in the supporting structure. The structural component functions to ensure the survival of the system from the environment (pictured as sun and cloud), i.e., provides the information carrier with positive feedback (Smiley face); otherwise, the information carrier will not be able to execute its “successful” sequence and the entire system will naturally perish. In the biological systems, the rule for information transfer to the structure is the genetic code or Watson-Crick base pairs where the protein sequence reflects the information of mRNA. Nothing can advance and adapt without Darwinian evolution; it is the most fundamental feature of life itself, i.e., “life” is defined as Darwinian evolution. (**b**) The universal definition of Darwinian evolution.

Biological systems as we know them transfer genetic information from the genome (DNA or RNA) to proteins or towards functional RNAs (e.g., rRNA, ribozymes, different regulatory small RNAs). The genetic code is the established “rule” that secures the correct translation from one type of polymer (RNA) to another type (protein). In the case of functional RNAs, the “rule” is just Watson-Crick base pairing. These two components: an information carrier and a functional executive component are described as genotype and phenotype, interacting in a feedback loop that maintains the genomic stability or “guiding” the outcome from genome variations [11]. The information coming from the DNA or RNA works in tandem with the structure carried by the protein or the structural/functional RNA sequence. If a mutation (a change of the information carrier) occurs, the transfer of information proceeds to the structural component “blindly”, governed only by the established rule (genetic code or base pairing). The altered system will then be subject to the process of natural selection and will either survive or perish (Figure 1a). If the mutation(s) survives, the genetic imprint will remain intact with an increased likelihood of propagating itself in subsequent generations. In this way, a beneficial mutation enables a biological system to persist in a particular environment. This adaptive property is at the core of Darwinian evolution (Figure 1a “smiley face”). Had the surviving structure (even highly efficient in the specific milieu) been independent of its information source, it would not be able to repeat and secure its “success”. Without this link from the surviving structure to the informational source, Darwinian evolution would have been thwarted without advancing and the evolution to more complicated forms would naturally stop. This notion is important as the fundamental difference between Darwinian evolution and natural processes, where more complex or bigger structures (e.g., growing crystals or polymers) are firmly following just the laws of physics and chemistry. On the contrary, information based, dynamic selection could proceed in various directions (also called open ended), determined by the contribution of any specific new structure to survival. For this mechanism to flourish, a dynamic process must be established to secure the stability of the entire system.

The principle of “Dynamic kinetic stability of life”, in turn, addresses the inevitable degradation of living systems formally stipulated by the Second Law of Thermodynamics [14]. Biological constructs do not contradict the Second Law; biological processes only resist this tendency. For example, enzymatic RNA or DNA replication works as a counter to RNA or DNA degradation and DNA repair enzymes function against the loss of information stored in DNA. Enzymes evolve with the feedback provided by Darwinian evolution and any newly developed feature follows this pattern of evolutionary development. The activities that provide for this dynamic stability may include any biological process such as replication, transcription, translation, or even a “simple” process like RNA-peptide interactions. Yet, it must invariably direct the system toward survival. At the beginning of life, Darwinian evolution was the only process that could trigger the formation of more advanced and better adapted forms. This mechanism exists as a pillar of life as we know it. Therefore, life is properly identified with Darwinian evolution in every way. In summary, according to the arguments presented so far, a simple and universal definition of Darwinian evolution based on its mechanism of action becomes (Figure 1b):

*“Darwinian Evolution is a Dynamically Stable System, Where Information Determines Its Own Supporting Structure.”*

Information and structure/function are the integral components of this definition. Although the description is very simple, it possesses universal value and, in a sense, describes a self-organizing system with the ability to increase and adapt its complexity. The two components are working in tandem, recognizable through their interrelationship and the ensuing mutual consequences. In addition, the definition for Darwinian evolution does not include specific requirements for polymers or any other building blocks of information and structure; therefore, life could be realized naturally or artificially out of different components (e.g., electro-mechanic, synthetic polymers). Although the presented description of Darwinian evolution possesses minimal components and is easily understandable as a mechanism, the origin of this system is complicated and difficult to discover. Even so, some bold logical conclusions are discussed in the following section.

## 3. The Information, the Structure, and the Corresponding Rule Are Locked in Its Origin

How is it possible to bootstrap the components in Figure 1 and set Darwinian evolution in motion in natural conditions? All three components: the information, the structure/function, and the rule between them are mutually dependent for their existence, i.e., the components are attributes of each other and cannot be analyzed independently. If we take the information, for example, it cannot exist without resolving the structure/function component, otherwise, the string of units (e.g., nucleotides of RNA) would be random and without information. It would be like the working of a mechanical clock without installed hands: you would never know what time it is. In order for the information to determine the structural/functional component, it needs a rule to execute the process of transfer of the information, i.e., genetic code or Watson-Crick base-pair, therefore the rule should exist also with the information and with the structure/function. It is evident that the three components cannot originate independently and must be established in the same physicochemical set of processes. The assembly of information cannot arise separately in another system different from the existing one. It is obvious that all components from Figure 1 (information, structure, and the rule for their interactions) are “locked” and inter-reliant in their origin and must be formed within the same group of processes and within the same timeframe. In that case, based on the mutual existence of all components given in Figure 1, a logical conclusion for the beginning of Darwinian evolution and life should be:

*“During Origin of Life, the Information, the Structure, and the Corresponding Rule Are Locked in Their Origin”.*

By accepting the law for the mutual interdependence of information-structure/function, a logical solution for “the chicken or egg” problem emerges. Over the years, the “chicken or egg” problem was formulated as an effort to seek what comes first: RNA or peptides/proteins, i.e., information or the structure? According to Figure 1 and following the law, we should not ask that question because there is no possible answer in a way as it is verbalized, i.e., both come first, RNA and peptides/proteins. The modified variant of the “chicken or egg” problem would be: How do RNA and peptides/protein become interdependent in order to provide a primitive translation?

### 3.1. The Beginning of the Biological Information

All biological systems utilize information on different levels and are embedded in different forms. There is an interconnection and a mutual dependence from bottom to top (e.g., from genetic to epigenetic to neurotransmission and pathways) and from top to bottom (e.g., from epigenetic toward genetic and metabolic) in a global informational network [15,16]. In the beginning, we are expecting one type of information to form a Darwinian evolution pathway. Paul Davies of Arizona State has emphasized information as a major component of life. Together with Sarah Walker, he published a definition based on the concept that life could be defined as information management (or control).

“As we have presented it here, the key distinction between the origin of life and other ‘emergent’ transitions is the onset of distributed information control, enabling context-dependent causation, where an abstract and non-physical, systemic entity (algorithmic information) effectively becomes a causal agent capable of manipulating its material substrate” [17].

Although an information repository is an attribute of life and certainly needs management, it is not clear what force(s) establishes this relationship. Therefore, “information management” needs a mechanism to determine its implementation and without it, the definition is incomplete. The storage and propagation of information may change during the origin of life, but this does not explain the difference between chemical and biological entity, therefore it is not a unique feature of life. The mechanism for the transition of informational storage is rooted in the process of Darwinian evolution itself. However, Paul Davies and Sarah Walker (and others) stipulate that Darwinian evolution is somehow separate and insufficient to distinguish living from nonliving matter, although it “still drives” the separation between the two.

“While we have stressed that Darwinian evolution lacks a capacity to elucidate the physical mechanisms underlying the transition from non-life to life or to distinguish non-living from living, evolution of some sort must still drive this transition (even if it does not define it)” [17].

Looking at what constitutes Darwinian evolution, we naturally reach the opposite conclusion. Darwinian evolution has the innate ability to define the transition from non-life to life. The provided (Figure 1) mechanism for Darwinian evolution shows a clear difference between an information-based system capable of evolving and adapting from all other physicochemical systems. Thus, the process of Darwinian evolution and “life” are the same phenomenon.

### 3.2. The Beginning of the Biological Structure

According to the mutual interdependence for the origin of Darwinian evolution described above, the formation of a biological structure should come together with the corresponding source of information. In a prebiotic milieu, many structures (e.g., short peptides, short RNAs, lipid vesicles, micro-chambers, gradients, the network of chemical and physical interactions) are able to generate suitable conditions for life to emerge, but until information dependent feedback (Figure 1) appears, those will define the environment suitable for life and not life itself. Many hypotheses do not make a clear difference between a structural component supporting life and the structure inextricably bound to Darwinian evolution, i.e., life.

The “metabolism first” hypothesis postulates that CO_2_ and NH_3_ fixation plus energy lead to the synthesis of amino acids and nucleotides. In that case, the building blocks may be synthesized but there is no mechanism showing how the structure will be generated by the informational source. The “lipid-world” [18,19,20], which is a modified variant of the “metabolism first” scenario, also does not provide a mechanism for the formation of these interactions according to Figure 1. In this hypothesis, lipid vesicles provide suitable conditions for proton gradients to reduce CO_2_ and trigger an organic production line to drive proto-metabolism for some of the basic biochemical components necessary for life [21,22]. Another scenario describes lipid lamellae on the earth’s surface with the potential to trap polymers (e.g., short RNA and peptides) undergoing a selection process accompanied by dry/wet cycles. The assumption of the gradual emergence of Darwinian evolution within multiple networks of lipid vesicles based on polymer interactions is presented as the next step but again, no mechanism is provided [2,23]. Lipid vesicles or lamellae may sustain integrity, but positive feedback from the vesicle structure to an information source capable of determining the synthesis of the same stable lipid vesicles is unspecified. Newly formed vesicles or lamellae, even if they contribute to the assembly of polymers, would not be able to advance further without a link to the informational source (see Figure 1), i.e., lamellae formation would occur with the same probability for every new cycle driven entirely by physicochemical laws. It is tempting to picture a scenario where polymers capable of utilizing Darwinian evolution hijack the lipid vesicles or a lamellae system, thus “organizing” a primitive biochemistry constituting a suitable environment for proto-cell formation. According to this scenario, some collaborative research for vesicle formation and polymer (e.g., RNA, peptide) interactions should be encouraged.

One of the aims of the origin of life research is to provide experimental evidence for the abiotic formation of primitive translation. However, if the hypothesis does not suggest a mechanism for the initiation of Darwinian evolution, it is unlikely to be a successful research avenue. Usually, ribozymes, synthesized under laboratory conditions that resemble natural protein-enzyme processes are considered crucial to the origin of life. Random aminoacylation performed by a ribozyme is insufficient to explain the origin of Darwinian evolution because positive feedback from the resulting peptide(s) to the ribozyme does not exist. Such feedback necessarily requires sequence-specific aminoacylation and it is hard to imagine how this capability would appear in a ribozyme. Further, non-specific peptide synthesis, even if it could generally increase the stability of the ribozyme (complex), will not provide any reproducible selective advantage [24,25,26,27]. Theoretically, RNA self-replicating ribozymes could trigger Darwinian evolution when information and structure/function are carried out by the same RNA, but the natural formation of this type of ribozyme is highly unlikely as we will discuss later. Another hypothesis describes peptides as the first important entities for the origin of life, before RNA or DNA, and the code formation [28,29,30,31]. The peptide-first type of hypothesis faces the same problem for initiating Darwinian evolution as was described for the metabolism-first or lipids-first suggestions. Without a doubt, short peptides could be directly synthesized in most of the proposed prebiotic scenarios and some amino acid combinations would have a significant functional impact [28,29,30,31]. Despite this possibility, if the peptides are not coded, Darwinian evolution cannot exist and the system will not advance toward longer functional peptides, i.e., the formation of functional peptides would occur with the same probability as those that were initially synthesized. On the contrary, if we can describe the formation of short peptides that are coded, then the successful peptides would be repeated more frequently and would allow for the development of longer functional peptides.

A possible way for the development of Darwinian evolution was suggested by Burton and colleagues. In this scenario, the interaction between the information carrier (RNA) and the structural component (peptide) does not occur by direct interactions, but it is only facilitated by stabilization from the surrounding membrane [32]. The authors assume that polyglycine could play an important role in stabilizing protocell membranes and this could trigger a selection force to preserve a system producing predominantly polyglycine. Similar to most bacteria walls containing peptidoglycan cross-linked as a UDP-MurNAc-pentapeptide polymer, the ancient polyglycine could generate a cross-linked membrane supporting scaffold. In fact, a peptide with five Gly (Gly5) is shown in *S. aureus* together with L-Ala and D-Gln. In other species (e.g., *E. coli* and *B. subtilis*), Gly is missing but L-Ala, D-Ala, and D-Gln are used, implying that glycine is not unique in this function [33]. Theoretically, in the described mechanism, polyglycine could provide positive feedback toward the information carrier and promote the formation of the genetic code. How suitable and specific glycine is for that particular function in comparison to other amino acids remains to be answered experimentally.

Poly-Gly could contribute through another activity to the prebiotic synthesis. Van der Gulik noticed that Gly-Gly di-peptide possesses amino acid polymerase activity and therefore could trigger a positive loop and Darwinian evolution [34,35,36,37]. In van der Gulik’s scenario, Gly-Gly may help the synthesis of an RNA replication peptide (usually containing Asp). In that case, a short RNA would remain stable longer in an environment where Gly-Gly would be coded. In this hypothesis, it is not very clear how the information embedded in RNA will determine a coded Gly (or any other amino acid) in a sequence-specific manner, i.e., how will the non-coded Gly-Gly and the RNA replication peptide become coded? The RNA information-carrying sequence must arise because of the Gly-Gly peptidyl-transferase function and must establish a sequence bias step in the chain of reactions from Gly-Gly and RNA replication peptide toward newly formed RNA. Also, since Gly-Gly (or modified variants) played an essential initial role in the synthesis of peptides, what is the selection force driving it toward a new way of protein synthesis by ribosome formation later in the evolution?

### 3.3. The Beginning of the Genetic Code

For life as we know it, the genetic code is the rule where the information embedded in RNA determines its own supporting structure (proteins) (Figure 1). According to the mutual interdependence of these components, the origin of the genetic code and the origin of Darwinian evolution must have happened within the same interlinked process and cannot be explained separately. There are many hypotheses attempting to describe the formation of the universal genetic code [38,39,40,41,42,43,44,45,46]. It is not in the scope of the current paper to describe and analyze these hypotheses in detail, but only to emphasize their relevance to the definition of Darwinian evolution as shown in Figure 1. According to some theories, the link between the codon and its cognate amino acid originates in a manner different from peptide or protein synthesis and only later adapts to the need for protein translation [40,42,44,47]. That concept postulates the existence of an initial “RNA-only” stage (e.g., RNA world) which requires further steps of development toward modern translational machinery. Despite many suggestions, there is no commonly accepted model without significant gaps.

Currently, there are two basic concepts for the stepwise evolution of the universal genetic code: the first type describes adding new coded amino acids as a function of an increasing number of meaningful nucleotides as part of the codon. In this case, every new meaningful codon nucleotide (out of three) brings more combinations and more coded amino acids. Usually, the increasing number of codon combinations of the newly assigned amino acids are shown in tables. The positioning of a new amino acid with a new codon(s) is dependent on a variety of different driving forces (e.g., coevolution, stereochemical, hydropathy). All 20 canonical amino acids and some non-canonical ones were assigned during the process of coevolution with the translation machinery in the direction of 3 nucleotide recognition [32,41,45,47,48,49,50,51,52,53]. Most hypotheses assume all four nucleotides (A, U, G, and C) to be available for novel codon combinations from the beginning of the genetic code. The second type of hypotheses takes a different approach. The three nucleotide (two determinative plus one wobble) codon system was developed earlier than the entire set of coded amino acids with only a few amino acids [46]. We postulated that the nucleotide codon combination for any amino acid was determined by two major factors: the available nucleotide combinations not taken yet by previously coded amino acids and the physicochemical properties of the newly coded amino acid that would improve the chances of survival of the system during the codon assignment. Initially, there were mostly GC-rich RNA available for codon formation, therefore the first assigned amino acids are predominantly with GC-rich codons. In that case, ordering the codons/anticodons from GC-rich toward AU-rich represents the chronological order of the amino acid assignment. If we align this order with the corresponding aminoacyl-tRNA synthetases (aaRS) for each amino acid, a very peculiar picture for the development of the genetic code and the corresponding aaRS emerged [46]. The observation shows a distinctive zonal distribution of aaRS for class II and class I along with the GC codon content. This remarkable correlation suggests at least two major periods of codon formation: pre-LUCA I and pre-LUCA-II. During pre-LUCA I, only the initial forms of class II aaRSs were developed with GC-rich codons. Later (during pre-LUCA II), class I aaRS begins its evolution with AU-rich codons along with the rest of class II. Pre-LUCA I was the time where the three-nucleotide coding translation apparatus was developed as a primitive structure with only few first coded amino acids [46]. Although all three nucleotides were part of the codon/anticodon recognition and were available, this logically would be a very primitive process with a low fidelity method of translation. During pre-LUCA I, the minor groove anticodon/codon recognition was developed mostly for the third and second nucleotide of the anticodon (or the first and second for the codon) due to the initial formation of proto-forms of helix 44 with A1492, and A1493 nucleotides as part of the small ribosomal subunit. During that time, the first anticodon nucleotide (the wobble one) would be very “leaky” and less meaningful because the G530 latching mechanism was not developed yet. In that case, the ribosome was not able to recognize cognate tRNA-aa complex from near-cognate tRNA-aa complex. The ribosome-tRNA machinery should be relatively small but sufficient to maintain the reliable transfer of the information from the primitive mRNAs/tRNAs toward short peptides. The high fidelity codon/anticodon recognition would be developed in the second pre-LUCA II period, where more advanced ribosomal loops and peptides/proteins would coevolve [54]. In pre-LUCA II, the ribosome would develop additional RNA structures like the sarcin-ricin loop (SRL) from large subunits, along with factors like EF-Tu and G530 “latching” conformation mechanism by which the fidelity and efficiency of the codon/anticodon recognition would dramatically increase. The upgraded ribosome would allow only the cognate tRNA-aa complex to proceed for peptide formation and would discriminate cognate from near-cognate one. At the same time, the first primitive RNA polymerase would increase its fidelity, populating more A/U nucleotides in the genome. The combination of increased A/U nucleotides in the RNA and the increased third nucleotide codon recognition opened the possibility for new codon combinations and amino acids assignment described in pre-LUCA II. In this hypothesis [46], the formation of the first codons is established as founding Darwinian evolution, i.e., only by the formation of coded amino acids did the system become more stable due to the newly formed positive feedback (Figure 1).

## 4. Too Many “Evolutions”: We Need Just One

Currently, the term “evolution” is used in almost every scientific field and largely refers to the increasing system’s complexity (e.g., evolution of the universe, evolution of the solar system, chemical evolution, pre-biotic evolution). When we describe the origin of life, the term “evolution” cannot be used only for an increase in complexity, but rather to emphasize the mechanism behind the process, otherwise, the difference between chemistry and biology cannot be appreciated and understood. Recently, Higgs published his model for the stages of evolution, the driving forces, and the selection process which led to the origin of life [9]. The stepwise increase in complexity is governed first by selection without replication, second by chemical evolution with replication, and finally, by biological evolution with replication and relatively large genomes. The key criterion for Higgs is the emergence of replication as a factor of stability that distinguishes the process of evolution (chemical and biological) from the initial chemical selection based on its physicochemical stability. At what stage does Darwinian evolution emerge according to Higgs?

“Darwinian evolution requires a mechanism of replication that passes on the properties of the parent, a mechanism of selection that allows fitter individuals to survive and/or reproduce faster than less fit individuals, and a mechanism for the generation of diversity in the population [9].”

Simply put, Darwinian evolution is replication with heredity and variations. Consequently, the chemical evolution described by Higgs is the same as Darwinian evolution since it is driven by replication. Biological evolution is also Darwinian evolution for the same reason, but at a higher level. Biological evolution is triggered by mutations in a larger genome which is modified without the need to be chemically synthesized from the environment.

If we take the definition for Darwinian evolution in Figure 1, we see that all elements presented by Higgs for Darwinian evolution are included (Figure 1a), but in a more comprehensive way. The difference is that the process of replication is not emphasized as the only stabilizing factor, but rather opens possibilities for additional factors (e.g., peptide/protein-nucleotide interactions, repair, metabolism which can lead to environmental independence) to be incorporated as well.

The next intriguing question is: where to place the boundary between chemistry and biology? This question is also tightly linked with the quest for a definition of life. Many scientists avoid this question as not important, thinking that it does not give us any experimental advantage toward the origin of life research [4]. The author and Higgs consider the definition of life in terms of the increase in our ability to differentiate life from non-life. For Higgs, the boundary is somewhere between chemical evolution and biological evolution [9]. The argument for his decision includes an open-ended scenario in biological evolution versus a progression in chemical evolution where the selection results in a more predictable fashion. Without a doubt, if we have a relatively simple system with a small genome (e.g., few meaningful nucleotides), the variants for adaptation are extremely limited and predetermined by the environmental conditions, making it close-ended. If, however, the system possesses a larger genome size (e.g., genes coding proteins with a variety of motifs), the adaptation will depend on the resulting mutations and combinations, causing an open-ended scenario. In that case, the evolution from close-ended toward open-ended is a consequence of the increased complexity of the system. It is difficult, however, to show when open-ended modifications start to play. It is quite possible that the boundary is very close to the “chemistry” at the level of a few amino acids or nucleotides. Theoretically, a quite simple polymer complex may act in an open-ended fashion, despite the having diminished adaptive potential. Depending on conditions, one additional nucleotide or one additional amino acid (which may be hydrophobic or hydrophilic, positively, or negatively charged) may affect the reason for the survival of the system. Close-ended versus open-ended is not a qualitative criterion because either way, it is driven by the same mechanism rather than by a quantitative leap toward a new state of matter. When we are looking for the boundary between chemistry and biology, we should look for a new qualitative level of organization utilizing an unbiased and objective approach. This criterion stipulates the presence of Darwinian evolution according to Figure 1, i.e., a system capable of an information-dependent increase in stability and complexity. This description dramatically differs from an increase in complexity based on physicochemical features alone. In this case, the boundary between chemistry and biology rests at the moment when Darwinian evolution emerges. “Life” is any system performing Darwinian evolution, even if it is quite simple and composed only of a few meaningful nucleotides or amino acids. Based on these arguments, it is suggested that Darwinian evolution and life are the same phenomenon and the definition in Figure 1 should be utilized as its expression in action.

## 5. The Existing Hypotheses for the Origin of Life in Respect to Darwinian Evolution

Scientific evidence for the origin of life points to an extremely early event that could have happened any time between 4.5–3.9 billion years ago. Most likely it occurred any time between 200 Myr to 800 Myr after the Earth’s formation [55,56]. We may never know how long it took for life to emerge and the specific conditions for its formation, however under the proper conditions, it could have emerged within a relatively short time frame (e.g., a hundred years). In that case, life most likely emerged many times in different variants until one specific complex established itself as the fittest. We can picture an alternative scenario where evidence of life or associated fossilized remnants does not exist for about 1 to 2 billion years after the Earth’s formation. Hypothetically, many scenarios for the origin of life are consistent if it occurred much later. A hypothesis like panspermia offers a more suitable explanation for the beginning of life on Earth. It is suitable also to consider the statistically rare event of polymer combinations, which need a very long time (1 to 2 billion years) to occur. These scenarios, however, became unlikely due to the fact that life began very early after Earth’s formation, suggesting that primordial Earth happened to be a place suitable for the endogenous origin of life at a very early moment. According to the arguments above, the origin of life is a highly likely and statistically possible event supporting hypotheses with simplicity and high probability.

Any reliable hypotheses for the origin of life should include a way for the inter-reliant origin of information, structure, and the corresponding rule in a statistically relevant manner. The key to the origin of life research is to demonstrate experimentally how that could happen. Indeed, most of the suggested scenarios and experimental approaches include in one way or another the emergence of Darwinian evolution. However, most of the time, the moment of initiation of Darwinian evolution is not in the scope of these hypotheses, nor is a deeper mechanism provided. Currently, there are numerous scenarios for the origin of life emphasizing different “first-appeared” events. Theoretical analyses of some scenarios with respect to Darwinian evolution were already published [10].

Here, the author would like to utilize the formulated mechanism described in Figure 1 for some of the origin of life ideas. In the list analyzed by Tessera, we find: Metabolism-first scenarios (few variants), Replication-first scenario (the RNA world hypothesis with some modifications but without RNA-peptide world scenarios), and the combined metabolism-replicator scenarios based on eight different criteria [10].

“Each model is evaluated according to the following questions: (1) What was the initial chemical substrate? (2) Are there experimental data supporting the main hypothesis? (3) What was the energy source? (4) Is the chiral question solved? (5) What is the ability of multiplication of the systems? (6) Is there any heredity? (7) Were there plausible site(s) where the initial chemical substrates might arise? (8) Is the evolutionary path plausible?” [10].

The author agrees with Tessera’s conclusions based on those criteria for each scenario, but the most important, “Is the evolutionary path plausible?”, could be answered in a more precise way following the mechanism described in Figure 1.

### 5.1. Systems Before Genetic Information

It has been proposed that self-assembled autocatalytic and autonomous networks of prebiotic chemical species could evolve to produce more stable systems [10,16,18,19,20,57,58,59,60,61,62]. Usually, those suggestions explain not only the prebiotic synthesis of RNA and amino acids, but the transfer of information and heredity as a necessary byproduct. In a prebiotic environment, different molecular components possess different functions toward proto-cell formation, organizing itself as an autonomous chemical system(s) capable of chemical evolution [63]. It was demonstrated that mutually catalytic systems (MCS) can emerge and could maintain some structural integrity with oscillatory patterns [64]. The described autocatalytic network of interactions may be highly beneficial for the formation of more stable polymers and primitive metabolism from which a Darwinian evolution may start [18,62]. However, is it possible to explain the emergence of an RNA/DNA/peptide/proteins type of Darwinian evolution in the light of autocatalytic organized pattern reactions which are not based on the same components?

Let us assume the formation of an autocatalytic network dependent on Darwinian evolution (Darwinian evolution #1) that is not based on components specific to life as we know it (RNA/DNA, peptides/proteins). Should the polymers/reactions of Darwinian evolution #1 pave the way to the second Darwinian evolution #2 based on familiar RNA/DNA/proteins? The problem with this approach is the transition from Darwinian evolution #1 to Darwinian evolution #2 based on completely different components for information and structure. Process #1 will utilize information carrier #1, making a positive loop with structure #1, which are both different from the #2. In that case, the shift from established information/structure #1 will require the *de novo* emergence of an information/structure system #2, where functional Darwinian evolution #1 facilitates the formation of Darwinian evolution #2. The selection forces require system #2 to benefit #1, otherwise, the hypothetical #2 is unlikely to appear dependent on #1. The reason for proposing #1 is to explain the origin of #2 because if #2 arises independently from #1, we do not need to propose the origin of #1. If system #2 originates out of #1, it should be a byproduct of #1 as well; therefore, according to Figure 1, the origin of life is described twice—the first “ignites” the second life formation which must form a Darwinian evolution step of its own. It is hard to explain the formation of system #2 when it is based on prior information and structures of different types because the selection forces have already established #1 as the well-adapted one. We must assume #2 to be a modified version of #1 since it is a part of the same selection mechanism. Therefore, there could not be a *de novo* switch from #1 to #2 based on completely different components. As a result, #1 and #2 are of similar nature and cannot be made from different components. In addition, there must be an explanation as to why Darwinian evolution #1 is lost if it is essential for the existence of #2? This would make the interplay between two different Darwinian systems extremely unlikely. It seems logical that we must start with only one type of Darwinian evolution, which is RNA/peptide or RNA/peptide-like based with or without non-canonical amino acids but fundamentally using the same types of polymers and mechanisms as are known in all organisms. As an example, on this account, DNA information carrier took over from RNA as the main information carrier. Both types of polymers mutually rearranged their functions and upgraded the way of storage and utilizing information. In this process, DNA replaces RNA as the main information carrier as a new more stable polymer. However, this is not a fundamentally different polymer but only a variation of RNA with the same basic principle of Watson-Crick base pair interactions. Also, RNA does not vanish but occupies its fundamentally important niche of functions. In the same way, we cannot expect the emergency of Darwinian evolution #2 from different type(s) of Darwinian evolution #1, but suitable upgrades without losing the previous one.

Similar problems occur if the system is initially based entirely on RNA-only Darwinian evolution which must evolve into an RNA/peptide system with the familiar genetic code. RNA-only Darwinian evolution is similar to evolution #1 discussed above and RNA/peptide evolution follows #2, with the same information source (RNA). Peter R Wills and Charles W Carter Jr have already provided a detailed mathematical model showing that the possible transition from RNA Coding World (RCW) to a Protein Coded World (PCW) is a highly unlikely event.

“Coding cannot be bootstrapped in the RCW because the dynamics of the RNA activity—replication of genetic information and catalysis of coding assignments—is completely autonomous. Coding self-organization based on feedback-constrained bootstrapping accelerated the exploration of sequence space and directed the search toward an optimal code.” Also, “Thus, the dynamics of the PCW entail a direct, very rapid, intrinsically generative pathway to a self-organized state of encoded information processing, whereas there is no such possibility inherent in the dynamics of the RCW” [7].

### 5.2. RNA World Scenario(s)

Scientists today tend to agree that RNA was established earlier and its evolution predated DNA in the very first living forms. At present, all organisms use the information stored in RNA in a variety of ways. The sequence of RNA may exist as specific 3D shapes with specific structural functions (e.g., rRNA) or could function as an enzyme (ribozymes) control gene expression to perform regulatory functions (e.g., miRNAs), or be translated into proteins (mRNA) in the ribosome where protein synthesis takes place. In all its functions, RNA demonstrates close dependence on proteins. RNA requires proteins to be synthesized (or replicated) and proteins require RNA to be coded. In view of this, the origin of life has taken the shape of “the chicken or the egg” dilemma. Which comes first: the RNA or proteins? Gilbert first published his “RNA world” hypothesis in 1986 [65] with the intention to solve the “the chicken or the egg” problem and to simplify the origin of life by eliminating proteins (or peptides) from initial events of life formation. His work assumed that ribozymes had an essential role in the early formation of life by simultaneously performing the role of information carrier and enzymatic functions without the need for proteins. The RNA world concept was motivated by recent discoveries of Thomas Cech and Sidney Altman (awarded the Nobel Prize in chemistry in 1986) of self-splicing rRNA and the ribonuclease P, respectively. These two discoveries showed RNA’s catalytic properties and gave inspiration to Gilbert to develop his hypothesis. The existence of ribozymes suggested that auxiliary proteins were unnecessary for Darwinian evolution to proceed. The RNA world is compatible with the described mechanism in Figure 1 but in that case, the information and the structure are based on the same type of polymer: RNA (or RNA-like). Thus the “chicken or the egg” dilemma appears to be solved. The RNA world hypothesis is now widely accepted as the most plausible explanation for the origin of life. Sulfur, phosphosphorus, and water from ancient volcanoes could create an acidic environment needed to maintain RNA stability in this primordial world [66]. Many synthetic ribozymes developed under laboratory conditions are able to perform a variety of enzymatic tasks including RNA self-replication [67,68]. Many naturally existing RNAs with different catalytic activities are considered relics from this ancient RNA world [69]. The hypothetical sequence of events leading to the emergence of the RNA world has been described [70]. Prebiotic chemistry leads to the formation of nucleotides, lipids, and amino acids, which can polymerize. The initial short RNAs or RNA-like molecules could maintain some of the sequences through a high fidelity non-enzymatic template-directed replication which should oppose their degradation. If there is no high fidelity pre-biotic non-enzymatic replication, the spontaneous formation of a self-replicating ribozyme is unlikely. This process is currently unknown, but the experiments in this direction continue. Difficulties for high fidelity non-enzymatic replication come from the fact that RNA tends to add mostly G or C ribonucleotides to the template but not A and U [70,71]. To avoid this “problem”, the search for all four nucleotides in high fidelity replication continues through a variety of approaches and the application of RNA-like templates (e.g., non-canonical RNA), showing better four nucleotide incorporation under specific conditions [72,73,74,75,76]. RNA maintenance is aided by the compartmentalization in the form of lipid layers or vesicles. After solving non-enzymatic replication, the next event(s) would be the emergence of longer intermediate forms due to RNA-RNA interactions. Naturally, by selection due to stability, a network of simple functional RNA oligos would generate a variety of ribozyme functions [77,78]. Eventually, a self-replicating RNA-replication ribozyme would emerge, which in fact will be the moment of initiation of Darwinian evolution. According to the RNA world hypothesis, the formation of the self-replicating ribozyme is the only way by which Darwinian evolution could start. The hypothesized self-replicator is the core and essential milestone for the RNA world hypothesis and has been the subject of extensive modeling with various conditions suggested for its existence [59,79]. It is important to note that the claim for the existence of a self-replicator is challenged by the RNA-peptide world hypothesis discussed later. Next evolutionary events include the emergence of the genetic code independent of aminoacyl tRNA synthetases (aaRSs). These would emerge later together with translation in which the information flows from RNA (or an RNA-like molecule) to coded peptides, i.e., the RNA world will undergo a polymer transition into the RNA/DNA/protein world. An alternative start for Darwinian evolution in the RNA world was suggested by Yarus [80] where a very simple NAD-like dinucleotide, the ancestor of a NAD cofactor, would contain nicotinamide and some of the canonical nucleotide bases. The resulting NAD-like molecules are called Initial Darwinian Ancestor (IDA) and would serve as a template to replicate the genuine 5′-5′ NAD or similar cofactors. This system would facilitate the formation of 5′-3′ RNA replication and the formation of ribozymes, i.e., an RNA world. In this scenario, the formation of Darwinian evolution in the RNA world would emerge much earlier than with the RNA-replication ribozyme due to the selection of IDA replication and IDA function as a primordial cofactor.

Although the antiquity of RNA is hard to dispute, the RNA world hypothesis is fraught with unresolved issues. High fidelity RNA replication is necessary to maintain ribozymes. Such high-fidelity replication could be carried out by an RNA replication ribozyme. This ribozyme is relatively long to arise by chance and therefore requires high fidelity non-enzymatic replication to produce an intermediate RNA (or RNA-like) precursor from which the replication ribozyme may be selected [72,73,75]. What are the driving forces that facilitate the transition from RNA-like high-fidelity non-enzymatic replication system to canonical RNA? The hypothetical RNA-like molecule capable of four nucleotide high fidelity replication is unlikely to evolve into canonical RNA if we assume that the RNA-like template already shows an advantage for better replication. The next transition also seems problematic. Established Darwinian evolution based on a self-replicating ribozyme (the replicator) should undergo a polymer transition to an amino acid coding system. In that process, a different type of selection pressure is needed where new polymer interactions must advance toward survival. The polymer transition is difficult to explain, despite many suggested scenarios [44]. Moreover, what is required is the selection of a totally different way of RNA or DNA replication by protein enzymes since the RNA replication ribozyme is already selected to perform high fidelity activity. As an example, today, we are observing high fidelity active ribozyme (the ribosome) performing a peptidyl transfer function; therefore, from this observation, it is reasonable to extrapolate that the initial self-replicating ribozyme could evolve into a variety of highly efficient replication enzymes for all replication activity today. In fact, there is no known naturally occurring self-replication ribozyme capable of replicating either RNA or DNA. These issues are not such that a reasonable explanation cannot be found, but are there hypotheses that avoid these caveats and provide a more plausible scenario? In addition, the RNA world hypothesis does not entirely avoid “the chicken and the egg” dilemma since it is difficult to explain the development of the genetic code and coded proteins. As was discussed above with Darwinian evolution #1 and #2, the polymer transition from RNA-only to an RNA-protein system is a very unlikely process. Gilbert initially proposed the RNA world hypothesis with the aim of making the origin of life simpler without the involvement of proteins or peptides. As a result, the RNA world hypothesis includes multi-step processes and has difficulties to adequately explain high fidelity non-enzymatic replication, self-replicating ribozymes, and polymer transition. These obstacles complicate the RNA world hypothesis and make it a less plausible scenario for the origin of life.

### 5.3. RNA-Peptide World Scenario

As was discussed above, the RNA world scenario has many issues, therefore an alternative model is needed. Maybe we should be looking for an interplay between RNA and short peptides to explain the formation of Darwinian evolution. Adapting the definition of Darwinian evolution from Figure 1b to life as we know it on planet Earth, we get:

*“Darwinian evolution is a dynamically stable system where the sequence of one hetero-polymer (e.g., RNA carrier of information) determines the sequence of another type of hetero-polymer (e.g., protein or peptide carrier of structure/function).”*

In summary, life begins when an RNA sequence establishes peptides that in turn interact and stabilize their encoding RNA. This process is known as “translation” and during the first steps when life emerged, it must have occurred in a simple system with a primitive tRNA, ribosome, and mRNA. In contemporary translation, the link between RNA and proteins is determined by proto-code where a specific tRNA (which carries a specific anticodon) must be aminoacylated with a specific amino acid providing the link between the information carrier and the structural component (Figure 1). This makes the aminoacylation of RNA “the most important chemical reaction in the universe”.

Since 2015, L.D. Williams, C.W. Carter, D. Caetano-Anollés and G. Caetano-Anollés, van der Gulik and D. Speijer, V Alva and AN Lupas, D. Kunnev and A. Gospodinov, S. Chatterjee and S. Yadav, C. Michel and J. Thompson, Didier Auboeuf, and others promoted new concepts for the origin of life as cooperation between RNA and peptides (RNA-peptide world) [11,13,30,46,81,82,83,84,85,86,87,88,89,90]. In the RNA-peptide scenarios, the information is carried by RNA (or RNA-like) polymer, but the structure comes initially from peptides and later from evolved proteins. In this hypothesis, “the chicken and the egg” dilemma is obviated by the cooperation between RNA and peptides in the process of initial primitive translation. In addition to RNA-peptide interactions, it seems that some amino acids can facilitate protocell formation due to the stabilization of fatty membranes [91]. If we accept the very first steps in the formation of Darwinian evolution in an RNA-peptide world, the translation, replication, selection of natural ribozymes, and the formation of the genetic code would necessarily follow [46,84]. In that case, the RNA-peptide world postulate that the components of Darwinian evolution—an information carrier, the rule (genetic code), and a function carrier emerged in the same set of physicochemical events as discussed above. Usually, the main criticism of the RNA-peptide world is the lack of replication, which is indeed critical to maintaining any newly formed Darwinian complex. Without a doubt, replication is vital for life, but according to the RNA-peptide world hypothesis, self-replication is not critical [46,84]. In the RNA-peptide world, however, self-replication of RNA is not required due to the ability of a short peptide to perform primitive replication [31,84]. In fact, the issue with replication is easily solved in the RNA-peptide world, which offers the possibility for the emergence of a simple peptide capable of holding two Mg^2+^ (or any suitable two-valence metal ions) at a specific distance and facilitates the beginning of enzymatic replication with a catalytic center specific for all modern DNA and RNA polymerases. Some authors advocate that the processes of replication and translation were initially the same [11,92]. In the author’s opinion, this is not necessary because within the very first steps of the formation of the Darwinian complex, non-enzymatic replication of any kind (even low fidelity) would be sufficient to maintain some short sequence motifs of RNA. Enzymatic RNA replication, i.e., performed by short peptides, must come from already coded amino acids, which would dramatically improve the stability of the system. Therefore, translation as a process comes just before the enzymatic replication but in a very primitive form. The self-replication of RNA never emerged during the origin of life nor at any time later in the evolution of the biological systems. In that scenario, Darwinian evolution is maintained around RNA-peptide dependent replication which we observe today.

The entity that carried out the very first primitive translation event is, in fact, the first life form and we can name it *“Terra Darwinia”*, which is the beginning of the Darwinian evolution. It would have contained few simple short RNAs sequences, with one or two meaningful nucleotides and the resulting peptide chain could have been comprised of just one or two meaningful amino acids [46,84]. RNA sequences longer than a few nucleotides and peptide chains longer than a few amino acids are not expected to emerge purely by chance, without the support of Darwinian selection. According to Figure 1, this simple interactive system contains positive feedback between the peptide chain and its associated RNA as an essential property of *Terra Darwinia*.

The very first steps towards the formation of primitive translation and primitive enzymatic replication are not experimentally demonstrated yet; nevertheless, the mutual cooperation between short peptides and short RNAs have been shown in systems where peptides are beneficial for specific aminoacylation, RNA stabilization, or RNA polymerization [31,93,94,95]. In addition, short RNAs can facilitate the process of peptide formation [96]. If we assume the mutually beneficial peptide/RNA role for the formation of the first primitive translation and the initiation of Darwinian evolution, the existence of vestigial motifs of the first genes and codons into modern rRNAs could be expected, and such evidence has been described [13,62,89,90,97].

A scenario that describes a possible mechanism for the initiation of the Darwinian evolution has been published [46,84]. According to this hypothesis, a relatively simple RNA-peptide system could emerge in hybridized RNA complexes due to primordial hot-cold, wet-dry cycles. Mutually stabilizing interactions between proto-peptides and RNA have been demonstrated experimentally, showing a significant increase in the thermal stability of RNA and an over 30-fold increase in peptide lifetime [95]. These data strongly increase the plausibility of RNA-peptide co-evolutionary models. The pre-biotic conditions could be very similar to those proposed by Baross and Martin [98] for “ribo-film formation”. In these conditions, processes of compartmentalization and concentration would occur for many organic components, including short RNAs or RNA-like polymers, amino acids, and lipids, as these would be trapped in a relatively stable biofilm, excluding to some extent the surrounding water. The biofilm could be deposited on the earth’s surface with different types of felsic and young sedimentary rocks and therefore, would be exposed to wet/dry and hot/warm cycles. Some of these RNAs would be aminoacylated and this could lead to the formation of short peptides due to the close proximity triggered by the RNA hybridization process [84]. A few of these hybridization dependent peptides would possess the ability to interact with hybridized RNAs in their native complex. A small subset of these peptides (called bridge peptides) would facilitate the specific aminoacylation of a new generation of RNAs with a similar sequence. These bridge peptides would naturally bias RNA aminoacylation and this interaction would be maintained. The resulting positive feedback from the bridge peptide to its cognate RNA would reinforce the stability of RNA peptide complexes, increasing the chance for the synthesis of more bridge peptides. This would pave the way to the process of specific aminoacylation, with the “correct” amino acid leading to the formation of a simple Darwinian evolution feedback process [46,84] of organic precursors of life becoming living forms endowed with a rich evolutionary path.

In summary, nowadays, many hypotheses are on the table for future experimental validation. Frequently, however, some of those hypotheses are “built” by just arranging the events from simple to complex with no emphasis on the genesis of Darwinian evolution. Most of the time, the hypotheses do not contradict each other, but are simply rearranged in a new way. Do those modified variants of already existing concepts offer a new vision for the origin of life? Without positive feedback from the structure/function to its information repository, Darwinian evolution cannot be initiated and therefore, such scenarios are not a suitable description for the origin of life but describe only the pre-biotic conditions suitable for life.
